# Direct superficial temporal artery access for middle meningeal artery embolization

**DOI:** 10.1177/15910199231225832

**Published:** 2024-01-09

**Authors:** Yang Qiao, Yi Jonathan Zhang, Samuel Tsappidi, Tej I Mehta, Ferdinand K Hui

**Affiliations:** 1 Department of Diagnostic and Interventional Imaging, 12340 The University of Texas Health Science Center at Houston, Houston, TX, USA; 2 Department of Interventional Radiology, 4002 The University of Texas MD Anderson Cancer Center, Houston, TX, USA; 3 Department of Neurointerventional Surgery, The Queen's Health System, Honolulu, HI, USA; 4 Department of Radiology and Radiological Science, 1501 Johns Hopkins Medicine, Baltimore, MD, USA

**Keywords:** Alternative access, superficial temporal artery access, middle meningeal artery embolization, chronic subdural hematoma, onyx

## Abstract

Middle meningeal artery embolization has become an important option in the management of subdural hemorrhages with multiple prospective studies demonstrating efficacy and randomized controlled trial data on the way. Access to the middle meningeal artery is usually achieved via the external carotid artery to the internal maxillary artery, then the middle meningeal artery. We report a case where a patient with symptomatic left-sided chronic subdural hemorrhage also had an external carotid artery occlusion. Direct puncture of the superficial temporal artery allowed retrograde access to the internal maxillary artery and thus the middle meningeal artery. Successful embolization of the vessel with 1:9 nBCA was performed with near total resorption of the subdural collection by 1 month postprocedure.

## Background

Middle meningeal artery (MMA) embolization for chronic subdural hemorrhage (cSDH) was first described by Mandai et al. in 2000.^1^ Subsequent experience has demonstrated effectiveness in reducing the volume of cSDH in the absence or in combination with surgical evacuation. In patients where the external carotid artery (ECA) is occluded, routine access via radial or femoral puncture may not be feasible. This report describes a cSDH embolization performed via direct puncture of the superficial temporal artery (STA) to achieve MMA access and embolization.

## Methods

Retrospective analysis of the case log was performed in accordance with local IRB requirements with patient consent obtained. Case details and demographics were collected taking care to remove protected health information prior to publication. No conflicts of interest are present among authors.

## Case

**Figure 1.**
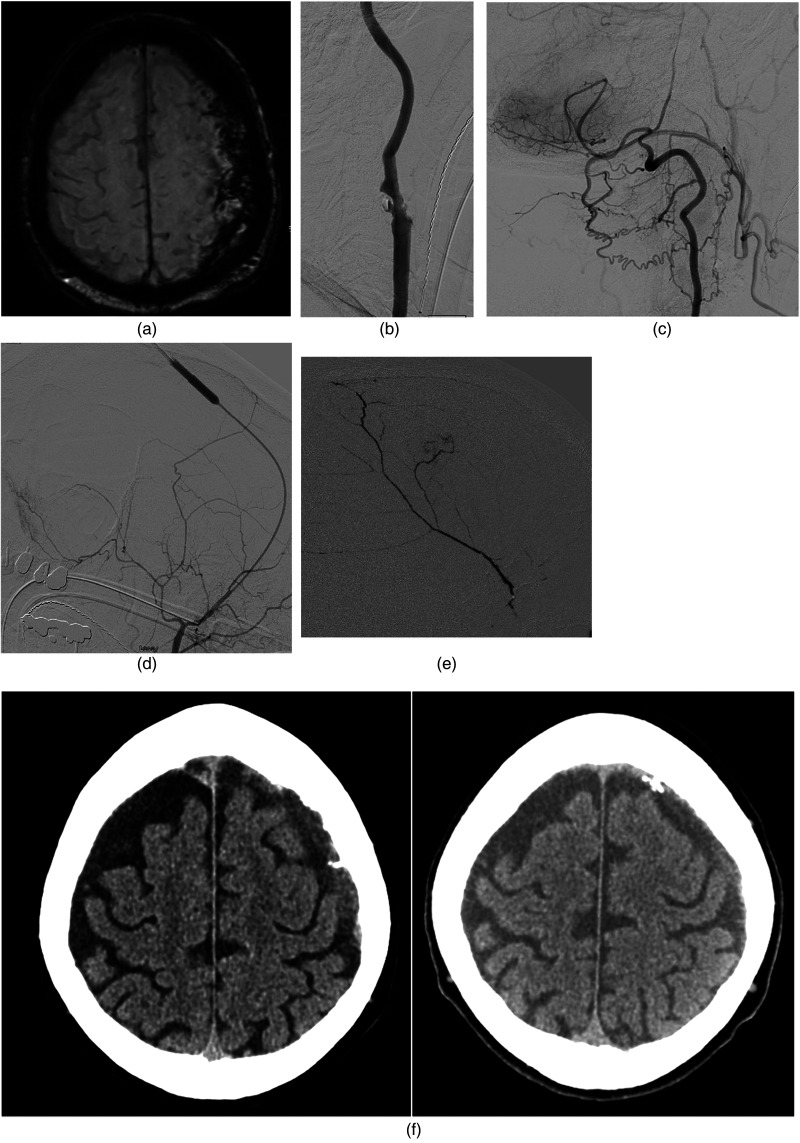
(a) Bilateral cSDH with a thick 2 cm left-sided cSDH was presumably the cause of neurological deficits via mass effect. (b) Complete occlusion of the left ECA precluded routine access to the MMA. (c) Angiography of the left vertebral artery revealed muscular collaterals feeding the left occipital artery which flowed in a retrograde fashion to fill the ECA territory, verifying patency of the STA and MMA. (d) Headway Duo 167 cm (Microvention) was advanced over an 0.014” microwire (Stryker) to reach the target location. (e) Successful right-sided MMA embolization with 9:1 nBCA (Cerenovus) via the Headway Duo 167 cm microcatheter (Microvention) was performed resulting in satisfactory penetration of the territory. (f) Follow-up CT 2 weeks (left image) and 1 month postprocedure (right image) revealed reduction of the cSDH to 8 and 5 mm in thickness. cSDH: chronic subdural hemorrhage; CT: computed tomography; ECA: external carotid artery; MMA: middle meningeal artery; STA: superficial temporal artery.

A patient in his 70 experienced a fall with right-sided hemiparesis activating a stroke code. Computed tomographic (CT) angiography and perfusion were performed revealing no perfusion deficit or large vessel occlusion, but instead bilateral cSDH with a thickness of 2 cm left-sided cSDH that was presumably the cause of neurological deficits via mass effect (Figure 1(a)). Due to a prior history of myocardial infarctions, systolic heart failure, hypertension, systemic lupus erythematosus, hemolytic anemia, and red blood cell antibodies, he was judged a poor surgical candidate for hematoma evacuation. An angiogram with intent to embolize the bilateral MMAs was performed with successful right-sided MMA embolization with 9:1 nBCA (Cerenovus Galway, Ireland) via a Headway Duo 167 cm microcatheter (Microvention, Aliso Viejo, CA). The left ECA was occluded (Figure 1(b)) precluding routine access. Angiography of the left vertebral artery revealed muscular collaterals that fed the left occipital artery which flowed in a retrograde fashion to fill the ECA territory, verifying patency of the STA and MMA (Figure 1(c)).

After an extensive multidisciplinary conference and discussion with the patient, the decision to perform direct puncture of the STA was made.

Using ultrasound guidance, a 4 French sheath, and micropuncture kit, the left STA was successfully accessed with injection showing flow into the internal maxillary artery and MMA. A Headway Duo 167 cm (Microvention, Aliso Viejo, CA) was advanced over an 0.014” microwire (Stryker, Kalamazoo, MI) to reach the target location (Figure 1(d)). Successful right-sided MMA embolization with 9:1 nBCA (Cerenovus Galway, Ireland) via the Headway Duo 167 cm microcatheter (Microvention, Aliso Viejo, CA) was then performed resulting in satisfactory penetration of the territory (Figure 1(e)).

Follow-up CT 2 weeks and 1 month postprocedure revealed reduction of the cSDH to 8 and 5 mm in thickness (Figure 1(f)), respectively; and complete resolution of neurological symptoms at 1 month.

## Discussion

MMA embolization continues to demonstrate robust and sustained evidence for the successful treatment of cSDH over time, whether in combination with surgical evacuation, alone, or in salvage cases of re-accumulation refractory to prior evacuation.^1–6^ Traditional standard access for MMA embolization is via femoral arterial puncture, with radial artery access now increasingly accepted as an alternative due to its improved safety profile in precluding retroperitoneal hematoma development. Although these established access routes are familiar to neurointerventionalists, occlusion at any point along the way to the MMA precludes successful embolization.

As demonstrated in this case report, direct access of distal ECA branches can provide an alternative pathway to the MMA in cases of proximal occlusion. Larger caliber superficial branches of the ECA with a gentle bifurcation angle with the internal maxillary artery, such as the superficial temporal or occipital arteries, provide ideal access candidates to help maintain maximal catheter purchase. Although direct STA access has been previously documented for embolization of a scalp arteriovenous fistula,^7^ this case is the first to demonstrate the technical feasibility of retrograde STA access for successful cSDH embolization. Careful patient selection, technical expertise, and extensive patient education should be provided prior to consideration of this approach. Close clinical follow up with low threshold for cross-sectional imaging is advised to monitor for any suspected postprocedure complication related to STA access.

Given variant anatomy of individuals and the vascular collateralization that exists in the setting of chronic occlusions, possible additional alternative routes for MMA embolization include access via the posterior circulation in select patient populations with patent vertebral-occipital artery communication. However, this approach may pose significant technical challenges in maintaining purchase secondary to tortuosity inherent to collateral vasculature. Another possibility includes direct puncture of the proximal MMA.^8^ however due to risks for the development of acute intracranial hemorrhage, this technique may not be conventionally advised.

## Conclusion

Pre-existing vascular abnormalities may preclude normative access to the MMA for patients with symptomatic subdural collections. The STA may provide an alternative access point for the MMA. This case report demonstrates the proof-of-concept, as well as its safety and efficacy.
